# Postural Complexity during Listening in Young and Middle-Aged Adults

**DOI:** 10.3390/e24060762

**Published:** 2022-05-28

**Authors:** Charles Dane Napoli, Karen S. Helfer, Richard E. A. van Emmerik

**Affiliations:** 1 Department of Kinesiology, University of Massachusetts Amherst, Amherst, MA 01003, USA; dnapoli@umass.edu; 2 Department of Communication Disorders, University of Massachusetts Amherst, Amherst, MA 01003, USA; helfer@umass.edu

**Keywords:** posture, complexity, listening, aging, entropy

## Abstract

Postural behavior has traditionally been studied using linear assessments of stability (e.g., center of pressure ellipse area). While these assessments may provide valuable information, they neglect the nonlinear nature of the postural system and often lead to the conflation of variability with pathology. Moreover, assessing postural behavior in isolation or under otherwise unrealistic conditions may obscure the natural dynamics of the postural system. Alternatively, assessing postural complexity during ecologically valid tasks (e.g., conversing with others) may provide unique insight into the natural dynamics of the postural system across a wide array of temporal scales. Here, we assess postural complexity using Multiscale Sample Entropy in young and middle-aged adults during a listening task of varying degrees of difficulty. It was found that middle-aged adults exhibited greater postural complexity than did young adults, and that this age-related difference in postural complexity increased as a function of task difficulty. These results are inconsistent with the notion that aging is universally associated with a loss of complexity, and instead support the notion that age-related differences in complexity are task dependent.

## 1. Introduction

How might aging manifest in the dynamics of biological systems? One hypothesis that has received much attention in recent years is the *loss of complexity* hypothesis, which claims that the loss of function associated with aging is the result of a loss of complexity in the underlying physiological systems [1]. As aging is associated with an increase in the incidence and severity of falls, the complexity of the postural system is of particular relevance [2]. Moreover, as individuals rarely perform postural tasks in the absence of an additional task (e.g., conversing with others or manipulating nearby objects), this postural complexity should be measured under ecologically valid, multi-task conditions.

Traditionally, however, research on postural behavior has not had as its focus the complexity of the underlying physiological systems. Rather, research has focused primarily on quantifying the *stability* of the postural system, often during laboratory-specific paradigms. In assessing stability, linear assessments of the center of pressure (e.g., ellipse area) and relations of the center of mass to the base of support (e.g., proximity) have been widely studied [3]. While it is true that these measures may provide valuable information in certain contexts, inherent assumptions of linearity restrict the breadth and depth of information that might be gleaned. When measuring the ellipse area of the center of pressure, for example, the traditionally dominant perspective is that an increase in variability is alone indicative of a pathological system. Such a perspective ignores the nonlinear nature of the postural system and conflates variability with pathology [4].

A more modern approach is to study the *response* of the postural system to mechanical perturbation [5,6]. This approach is particularly attractive to those who wish to study the stability of the postural system, as stability is often defined as the resistance of a system to perturbation. This approach, however, is not without its own set of limitations. For example, as an external perturbation is a discrete event, the data obtained during such an experiment are limited, naturally, to a discrete response. Moreover, there is difficulty in scaling the degree of perturbation to be applied, as well as in mitigating the risks associated with mechanical perturbation. An alternative approach is to examine the behavior of the postural system while individuals perform perceptual tasks (i.e., tasks in which the primary objective is to perceive ecological information) during mechanically unperturbed stance. This approach, while not unique to the present study, may permit the examination of more natural postural behavior and provide clinically relevant information.

Importantly, the examination of postural behavior must extend beyond traditional assessments aimed at quantifying the stability of the postural system. An assessment of *complexity* may provide alternative insight into the dynamics of the postural system across a wide array of temporal scales and under a wide variety of conditions. Note that this distinction between stability and complexity is neither arbitrary nor is it meant to suggest that assessments of stability are unimportant. Rather, it is meant to offer a delineation between two distinct features of biological systems and to suggest that neither feature should be overlooked. As complexity, much like stability, is more frequently intuited than precisely defined, it is unsurprising that there is no widely accepted definition of complexity. Roughly, however, the complexity of a system may be thought of as the degree to which the system exhibits meaningful connectivity across spatial and temporal scales [1].

Various techniques have been proposed to measure the complexity of experimentally obtained signals. One such technique, *Sample Entropy*, provides an estimate of signal complexity by computing the probability that two sufficiently similar segments of equal length taken from a signal will *cease to remain* sufficiently similar when the length of each segment is increased by one sample [7]. It follows that the Sample Entropy of a purely periodic signal will be equal to zero, while the Sample Entropy of a complex, biophysical signal will be greater than zero. If, however, one computes the Sample Entropy of a signal generated purely by stochastic processes (e.g., white noise), the Sample Entropy of that signal will approach some maximal value despite a distinct *lack* of complexity. This inability of Sample Entropy to distinguish, reliably, between signals with complex structure and those without renders this approach insufficient when attempting to measure biophysical signal complexity.

Accordingly, Costa and colleagues developed the Multiscale Sample Entropy (MSE) analysis, which, unlike Sample Entropy, considers the *fractal* structure inherent in biological systems [8,9,10]. In this approach, a signal is first coarse-grained into a given number of signals pertaining to a given number of timescales. This coarse-graining procedure consists of partitioning the original signal into non-overlapping windows of a given number of samples, averaging the samples within each window, and concatenating each window as to reconstruct the coarse-grained time series. This procedure will yield one coarse-grained signal, or *scale*, per window length, with the original signal occupying scale one. From here, Sample Entropy can be computed across timescales, thereby providing a more robust measure of signal complexity.

Assessing such a measure of postural complexity while individuals engage with a listening task may provide unique insight into how the postural system is able to reorganize its degrees of freedom in response to increased perceptual demands. Existing literature on postural behavior during listening suggests that an increase in listening effort is associated with an increase in postural sway area, particularly for middle-aged adults [11]. While this increase in postural sway area is generally interpreted as a reduction in postural control resulting from the availability of fewer resources, it is not presently clear how aging impacts the underlying dynamics of the postural system during listening.

Here, we apply the MSE analysis to postural data obtained while young and middle-aged adults engaged with a listening task of varying degrees of difficulty. By assessing the complexity of postural behavior during such a task, insight into how the postural system is able to adapt to increased perceptual demands might be gained. Moreover, assessing the complexity of postural behavior across *physiologically relevant* timescales (e.g., those associated with physiological tremor) might serve to further elucidate age-related changes in complexity. It is expected that the complexity exhibited by the young adults will be greater than the complexity exhibited by the middle-aged adults, and that this difference will increase as a function of task difficulty.

## 2. Materials and Methods

### 2.1. Participants

As part of a larger study, 16 young adults (18 to 28 years of age; mean 22 years) and 16 middle-aged adults (48 to 64 years of age; mean 58 years) were recruited, with both groups being comprised of 11 females and 5 males. Note that data from one young participant and two middle-aged participants were corrupted during collection, resulting in groups of 15 and 14 being used for the present study. The young participants all had clinically normal hearing (pure-tone thresholds <20 dB HL from 250 to 8000 Hz), and all but two of the middle-aged participants had clinically normal hearing (pure-tone thresholds <30 dB HL) from 250 to 4000 Hz. Participants were recruited from the general population and were screened for visual, vestibular, and motor impairments prior to collection. For a full description of participant characteristics, see Helfer et al. [11]. All procedures were approved by the University of Massachusetts Amherst Institutional Review Board (IRB).

### 2.2. Procedures

Participants stood with their feet shoulder-width apart on a 40 × 60 cm piezoelectric force platform (Kistler Instruments Corporation, Amherst, NY, USA) while listening to and repeating pre-recorded sentences. These sentences were designed to have low predictability while being grammatically feasible (e.g., *Theo found the pink menu and the true item here)*. During the listening task, a loudspeaker was placed 1.2 m in front of the participants and was adjusted to the ear height of each participant. The loudspeaker simultaneously played both a *target* sentence (i.e., a sentence which the participants were instructed to repeat) as well as two *masking* sentences (i.e., sentences similar to the target sentence which the participants were instructed to ignore). These masking sentences were played from random starting points as to minimize any grammatical alignment of the masking and target sentences.

For the present study, three conditions were analyzed. The first condition consisted of participants standing on the force platform without any listening task. This condition, referred to as the baseline condition, served to provide the experimenters with the baseline postural dynamics of each participant. The remaining two conditions consisted of participants standing on the force platform while performing the listening task described above. The difficulty of the listening task was modified for these two conditions by adjusting the signal-to-noise ratio (SNR) of the stimuli. In the first of these remaining conditions, the 0 dB condition, the combined energy of the masking sentences was equal to that of the target sentence. In the final condition, the −6 dB condition, the combined energy of the masking sentences was 6 dB greater than that of the target sentence. The order of conditions was randomized for each participant, with each condition lasting approximately 80 s.

### 2.3. Data Analysis

Ground reaction forces were collected continuously during each condition at 100 Hz and a custom written MATLAB script (The MathWorks, Natick, MA, USA) was used to compute the center of pressure (CoP). A second-order, zero-lag, bandpass Butterworth digital filter (1–15 Hz) was then applied to the data based on a sensitivity analysis (see Appendix B).

**Figure 1:**
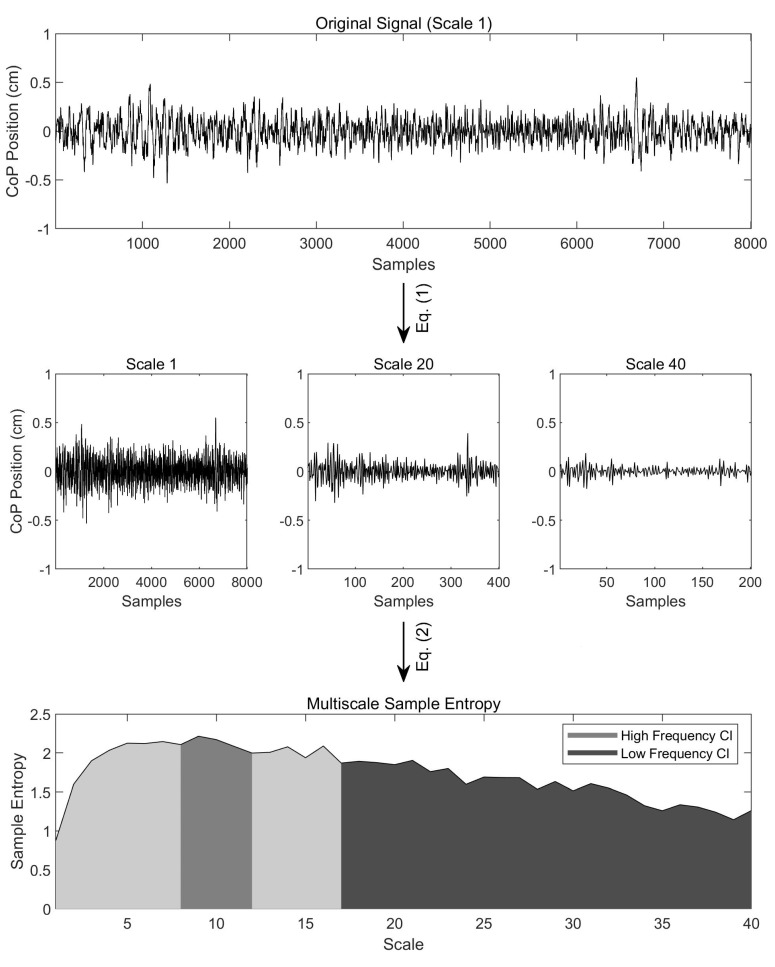
Multiscale Sample Entropy analysis performed on one representative CoP signal. First, the original signal is coarse-grained into a given number of signals by Equation (1). Then, Sample Entropy is computed for each of these coarse-grained signals by Equation (2). Complexity Indices are then computed by taking the sum of the Sample Entropy values within a given range of scales. Here, the high-frequency Complexity Index corresponds to 8–12 Hz (scales 8 to 12) and the low-frequency Complexity Index corresponds to 2.5–6 Hz (scales 17 to 40). Note that the area under the entire curve represents the overall Complexity Index, corresponding to 2.5–100 Hz.

Multiscale Sample Entropy was computed for the CoP data in the following manner (Figure 1). First, the original CoP time series was coarse-grained according to the following equation:

(1) yj(τ)=1τ∑i=(j−1)τ+1jτxi,  1≤j≤Nτ

where $y_{j}$ is a sample of the coarse-grained signal, τ is the scale, and $x_{i}$ is a sample of the original signal. This procedure was repeated for a total of 40 scales, with each scale greater than one yielding a new, coarse-grained signal. Sample Entropy was then computed for each of the 40 scales by the following equation:

(2) $$S_{E}\left(m,r,N\right)=-\ln\frac{\Phi^{m+1}\left(r\right)}{\Phi^{m}\left(r\right)}$$

where m is the number of samples being compared across segments of the signal, r is the radius of similarity, and N is the length of the time series. Here, $\Phi^{m}\left(r\right)$ is the probability that the segments will be sufficiently similar when comprised of m samples, and $\Phi^{m+1}\left(r\right)$ is the probability that the segments will be sufficiently similar when comprised of m+1 samples. Note that in the present study, r was set to 0.15 times the standard deviation of the signal and m was set to 2 [12].

After computing the Sample Entropy for each scale, Complexity Indices were computed by taking the sum of the Sample Entropy values within a given range of scales, as defined by the following equation:

(3) $$C_{I}=\overset{n}{\underset{\tau =b}{\sum }}S_{E}\left(\tau \right)$$

where $S_{E}$ is the Sample Entropy for scale τ and the Complexity Index is comprised of scales *b* through n. Here, three Complexity Indices were selected based on their corresponding timescales: an *overall* Complexity Index comprised of scales 1 through 40 (corresponding to 2.5–100 Hz), a *low-frequency* Complexity Index comprised of scales 17 to 40 (corresponding to 2.5–6 Hz), and a *high-frequency* Complexity Index comprised of scales 8 to 12 (corresponding to 8–12 Hz). Note that frequencies of 2.5–6 Hz are typically associated with voluntary movement, while frequencies of 8–12 Hz are typically associated with involuntary movement (e.g., physiological tremor).

### 2.4. Statistical Analysis

Two-way repeated measures analyses of variance (ANOVAs) with condition and age as within- and between-subjects factors, respectively, were performed for the three Complexity Indices (overall, low-frequency, and high-frequency) and for the two CoP directions (anteroposterior (A/P) and mediolateral (M/L)). Greenhouse–Geisser corrections were applied in any instance where the assumption of sphericity was violated (Mauchly’s W; *p* < 0.05). Post hoc pairwise comparisons were performed using *t*-tests with Bonferroni corrections. Effect sizes were reported in partial eta-squared (η^2^_p_) (0.01 = small; 0.06 = medium; 0.14 = large) for the ANOVAs and in Cohen’s *d* (0.20 = small; 0.50 = moderate; 0.80 = large) for the post hoc comparisons. A significance threshold of α = 0.05 was used for all inferential statistics. All statistical analyses were performed in JASP (JASP, Amsterdam, The Netherlands).

## 3. Results

### 3.1. Overall Complexity Index (A/P)

**Figure 2:**
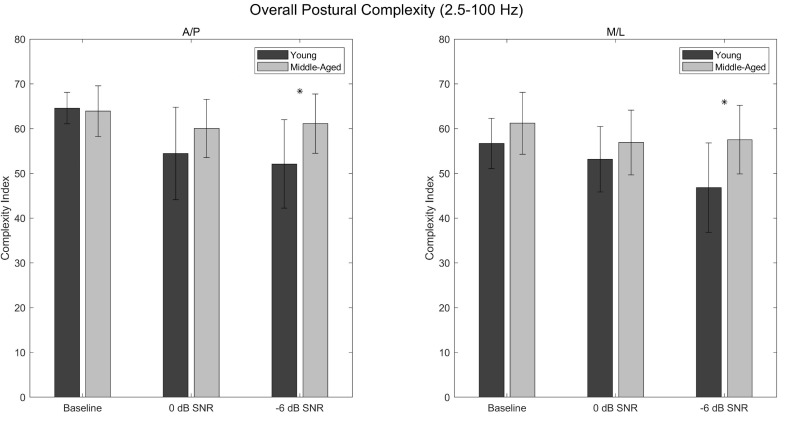
Overall postural complexity values corresponding to 2.5–100 Hz for the baseline, 0 dB, and −6 dB conditions. Anteroposterior complexity values are shown in the left panel, and mediolateral complexity values are shown in the right panel. Asterisks denote significant differences between the young and middle-aged participants. Error bars are ±standard deviation.

Main effects of condition (*p* < 0.001; η^2^_p_ = 0.311) and age (*p* = 0.025; η^2^_p_ = 0.173) were found for A/P postural complexity in the 2.5–100 Hz range, with postural complexity being greater in the middle-aged participants than in the young participants (Table A1 and Table A2; Figure 2). Post hoc analysis revealed that postural complexity was greater in the baseline condition than in the 0 dB (*p* < 0.001; d = 0.933) and −6 dB (*p* < 0.001; d = 1.020) conditions, with no significant difference existing between the 0 dB and −6 dB conditions (*p* = 1.000; d = 0.087) (Table A3; Figure 2). A significant condition × age interaction was also found (*p* = 0.034; η^2^_p_ = 0.131), with post hoc analysis revealing significant age-related differences in postural complexity in the −6 dB condition (*p* = 0.028; d = 1.201), but not in the 0 dB (*p* = 0.726; d = 0.746) or baseline (*p* = 1.000; d = −0.088) conditions (Table A1 and Table A4; Figure 2).

### 3.2. Overall Complexity Index (M/L)

Main effects of condition (*p* < 0.001; η^2^_p_ = 0.277) and age (*p* = 0.009; η^2^_p_ = 0.229) were found for M/L postural complexity in the 2.5–100 Hz range, with postural complexity being greater in the middle-aged participants than in the young participants (Table A5 and Table A6; Figure 2). Post hoc analysis revealed that postural complexity was greater in the baseline condition than in the 0 dB (*p* = 0.035; d = 0.515) and −6 dB (*p* < 0.001; d = 0.893) conditions, with no significant difference existing between the 0 dB and −6 dB conditions (*p* = 0.182; d = 0.378) (Table A7; Figure 2). A significant condition × age interaction was also found (*p* = 0.046; η^2^_p_ = 0.108), with post hoc analysis revealing significant age-related differences in postural complexity in the −6 dB condition (*p* = 0.005; d = 1.410), but not in the 0 dB (*p* = 1.000; d = 0.491) or baseline (*p* = 1.000; d = 0.595) conditions (Table A5 and Table A8; Figure 2).

### 3.3. Low-Frequency Complexity Index (A/P)

**Figure 3:**
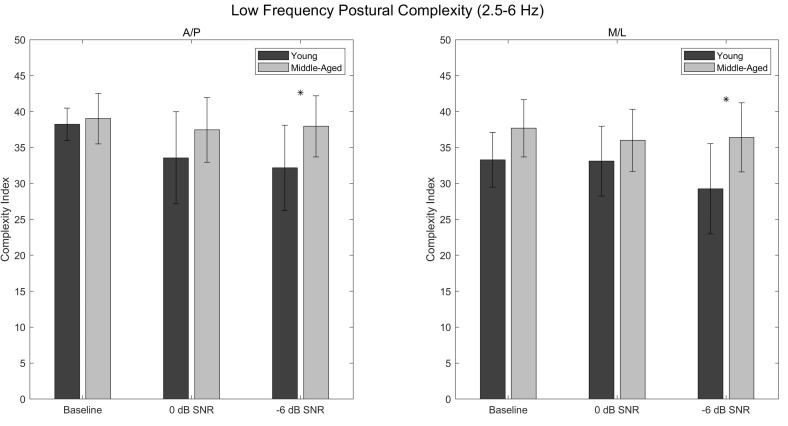
Low-frequency postural complexity values corresponding to 2.5–6 Hz for the baseline, 0 dB, and −6 dB conditions. Anteroposterior complexity values are shown in the left panel, and mediolateral complexity values are shown in the right panel. Asterisks denote significant differences between the young and middle-aged participants. Error bars are ±standard deviation.

Main effects of condition (*p* = 0.005; η^2^_p_ = 0.203) and age (*p* = 0.010; η^2^_p_ = 0.220) were found for A/P postural complexity in the 2.5–6 Hz range, with postural complexity being greater in the middle-aged participants than in the young participants (Table A9 and Table A10; Figure 3). Post hoc analysis revealed that postural complexity was greater in the baseline condition than in the 0 dB (*p* = 0.013; d = 0.661) and −6 dB (*p* = 0.004; d = 0.758) conditions, with no significant difference existing between the 0 dB and −6 dB conditions (*p* = 1.000; d = 0.097) (Table A11; Figure 3). While no significant condition × age interaction was found (*p* = 0.080; η^2^_p_ = 0.096), post hoc analysis revealed significant age-related differences in postural complexity in the −6 dB condition (*p* = 0.023; d = 1.227), but not in the 0 dB (*p* = 0.441; d = 0.827) or baseline (*p* = 1.000; d = 0.172) conditions (Table A9 and Table A12; Figure 3).

### 3.4. Low-Frequency Complexity Index (M/L)

Main effects of condition (*p* = 0.017; η^2^_p_ = 0.140) and age (*p* = 0.002; η^2^_p_ = 0.297) were found for M/L postural complexity in the 2.5–6 Hz range, with postural complexity being greater in the middle-aged participants than in the young participants (Table A13 and Table A14; Figure 3). Post hoc analysis revealed that postural complexity was greater in the baseline condition than in the −6 dB condition (*p* = 0.015; d = 0.557), with no significant difference existing between the baseline and 0 dB conditions (*p* = 0.921; d = 0.197) or between the 0 dB and −6 dB conditions (*p* = 0.192; d = 0.361) (Table A15; Figure 3). While no significant condition × age interaction was found (*p* = 0.068; η^2^_p_ = 0.095), post hoc analysis revealed significant age-related differences in postural complexity in the −6 dB condition (*p* = 0.002; d = 1.503), but not in the 0 dB (*p* = 1.000; d = 0.607) or baseline (*p* = 0.239; d = 0.924) conditions (Table A13 and Table A16; Figure 3).

### 3.5. High-Frequency Complexity Index (A/P)

**Figure 4:**
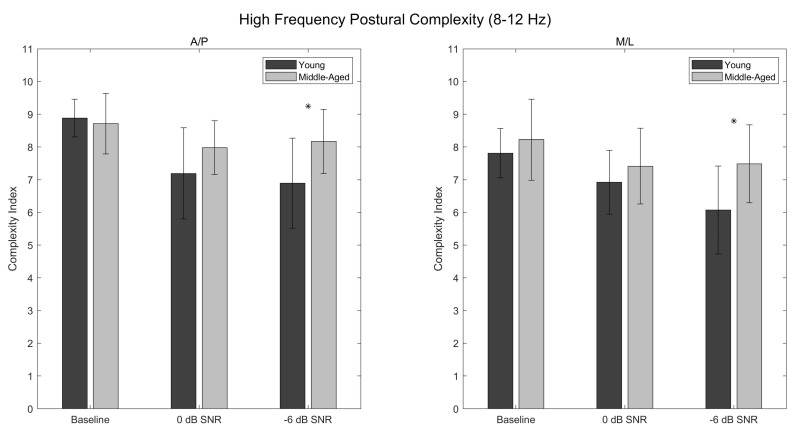
High-frequency postural complexity values corresponding to 8–12 Hz for the baseline, 0 dB, and −6 dB conditions. Anteroposterior complexity values are shown in the left panel, and mediolateral complexity values are shown in the right panel. Asterisks denote significant differences between the young and middle-aged participants. Error bars are ±standard deviation.

Main effects of condition (*p* < 0.001; η^2^_p_ = 0.407) and age (*p* = 0.035; η^2^_p_ = 0.155) were found for A/P postural complexity in the 8–12 Hz range, with postural complexity being greater in the middle-aged participants than in the young participants (Table A17 and Table A18; Figure 4). Post hoc analysis revealed that postural complexity was greater in the baseline condition than in the 0 dB (*p* < 0.001; d = 1.147) and −6 dB (*p* < 0.001; d = 1.197) conditions, with no significant difference existing between the 0 dB and −6 dB conditions (*p* = 1.000; d = 0.050) (Table A19; Figure 4). A significant condition × age interaction was also found (*p* = 0.020; η^2^_p_ = 0.153), with post hoc analysis revealing significant age-related differences in postural complexity in the −6 dB condition (*p* = 0.027; d = 1.205), but not in the 0 dB (*p* = 0.737; d = 0.744) or baseline (*p* = 1.000; d = −0.157) conditions (Table A17 and Table A20; Figure 4).

### 3.6. High-Frequency Complexity Index (M/L)

Main effects of condition (*p* < 0.001; η^2^_p_ = 0.394) and age (*p* = 0.030; η^2^_p_ = 0.163) were found for M/L postural complexity in the 8–12 Hz range, with postural complexity being greater in the middle-aged participants than in the young participants (Table A21 and Table A22; Figure 4). Post hoc analysis revealed that postural complexity was greater in the baseline condition than in the 0 dB (*p* < 0.001; d = 0.754) and −6 dB (*p* < 0.001; d = 1.102) conditions, with no significant difference existing between the 0 dB and −6 dB conditions (*p* = 0.218; d = 0.348) (Table A23; Figure 4). While no significant condition × age interaction was found (*p* = 0.054; η^2^_p_ = 0.112), post hoc analysis revealed significant age-related differences in postural complexity in the −6 dB condition (*p* = 0.020; d = 1.259), but not in the 0 dB (*p* = 1.000; d = 0.437) or baseline (*p* = 1.000; d = 0.366) conditions (Table A21 and Table A24; Figure 4).

## 4. Discussion

The present study assessed the complexity of postural behavior exhibited by young and middle-aged participants during a listening task of varying degrees of difficulty. Consistent with the loss of complexity hypothesis, it was expected that the young participants would exhibit greater postural complexity than the middle-aged participants, and that this difference would increase as a function of task difficulty. In comparing the postural complexity exhibited by the two groups of participants, however, the opposite result was observed; the middle-aged participants exhibited *greater* postural complexity across all Complexity Indices and across both CoP directions. Moreover, this difference appeared to increase as a function of task difficulty, with significant age-related differences in postural complexity emerging consistently in the −6 dB condition while not being present in the baseline or 0 dB conditions.

While these results were not expected, they are not entirely without precedent. In 2007, Costa et al. [13] found that postural complexity did not differ significantly between young and older individuals during quiet standing, provided the individuals in the latter group were of sufficient health (i.e., had no history of falling). Rather, it was found that healthy young and healthy older individuals both exhibited greater postural complexity than did older individuals with a history of falling. As the middle-aged participants in the present study were screened for visual, vestibular, and motor impairments, it is reasonable that no significant age-related differences in postural complexity were observed in the baseline or 0 dB conditions. Indeed, these participants were first recruited by Helfer et al. [11] in an effort to investigate age-related changes in postural control and speech perception that may appear despite a lack of associated impairments.

Additionally, Duarte and Sternad [14] found that older individuals exhibited greater postural complexity than did young adults during prolonged standing. Importantly, however, these age-related differences were found to depend upon the radius of similarity, r, expressed in Equation (2). As prolonged standing is accompanied by natural postural adjustments (e.g., shifting one’s weight), the resulting signal often contains a substantial number of outliers. If one group performs more frequent postural adjustments, these outliers may lead to differences in signal complexity, as *r* is typically multiplied by the standard deviation of the signal. When Duarte and Sternad corrected for these outliers by using a fixed *r* value, these age-related differences in postural complexity were no longer found.

Does such a finding by Duarte and Sternad suggest that the age-related differences in the present study are similarly dependent upon the radius of similarity (*r*)? First, one must consider the nature of the postural tasks in question. While the prolonged standing task implemented by Duarte and Sternad consisted of unconstrained standing lasting thirty minutes, the postural task in the present study lasted only eighty seconds. It is therefore unlikely that the postural data analyzed in the present study contained a similar number of outliers. Moreover, the standard deviations in the present study were, on average, greater in the middle-aged participants [11]. As a greater standard deviation increases the radius of similarity, using a fixed *r* value in the present study would serve only to *increase* the relative degree of postural complexity observed in the middle-aged participants.

How, then, might one reconcile the nature of these differences with the existing literature on postural complexity and aging? As significant age-related differences in postural complexity emerged consistently in the −6 dB condition, it is possible that the two groups of participants adopted two distinct strategies in response to the listening task and that these strategies became distinguishable only under more difficult acoustic conditions. If, for example, maintaining upright posture while engaging with the listening task proved more difficult for the middle-aged participants, these participants may have been unable to incur any reduction in the degrees of freedom being used for postural control [15]. In contrast, the young participants may have prioritized the perception of auditory information, even if this prioritization resulted in such a reduction in the degrees of freedom. This explanation is further supported by the finding that the postural complexity exhibited by the middle-aged participants did not differ between conditions, while the postural complexity exhibited by the young participants appeared to decrease as a function of task difficulty (Figure 2, Figure 3 and Figure 4).

Returning to the first study conducted by Helfer et al. [11], a moderate reduction in listening task performance was observed between the young and middle-aged participants. While this reduction may have been the result of early age-related changes in hearing, it is consistent with the notion that individuals in the latter group may have been unable to prioritize the perception of auditory information. Helfer et al. also reported a moderate increase in the 95% confidence ellipse of the CoP between the young and middle-aged participants during the listening task, further suggesting that maintaining upright posture while engaging with the listening task was more difficult for the middle-aged participants. These results, when combined with those of the present study, suggest that early aging may be accompanied by a decrease in the ability of individuals to regulate their CoP while conversing with others.

One additional finding is that the age-related differences in postural complexity appeared consistent across the three Complexity Indices. Within each Complexity Index, middle-aged participants exhibited significantly greater postural complexity in both the A/P and M/L directions than did young adults in the −6 dB condition (Figure 2, Figure 3 and Figure 4). Likewise, moderate-to-large effect sizes were found within each Complexity Index in the 0 dB condition, suggesting that the middle-aged participants exhibited greater A/P postural complexity than did the young participants, though these differences did not reach statistical significance. Note that direct comparison of postural complexity *between* Complexity Indices is not possible given the different number of scales within each Complexity Index (e.g., the overall Complexity Index is comprised of 40 scales while the high-frequency Complexity Index is comprised of only five scales).

While the results presented here provide unique insight into the dynamics of the postural system during listening, there are several limitations that should be noted. Firstly, participants in the present study were restricted to quiet standing, which may not be reflective of the multitude of postures individuals adopt while conversing. Secondly, the physical activity levels of the participants were not assessed despite these levels being potentially relevant to the interpretation of our results. Lastly, as noted by Helfer et al. [11], the baseline condition was measured in relative silence, which may have altered postural behavior by rendering participants unable to orient themselves towards a reference signal in the surrounding environment [16].

Overall, the results of the present study do not support the loss of complexity hypothesis put forth by Lipsitz and Goldberger [1] but rather the notion that age-related differences in complexity are task dependent [15]. Specifically, it was found that the postural complexity exhibited by young and middle-aged adults may not differ significantly during quiet standing if the individuals in the latter group are of sufficient health. Moreover, it was found that middle-aged adults may exhibit *greater* postural complexity than young adults when quiet standing is coupled with a perceptual task. These results should serve to caution against universally associating the degree of complexity exhibited by a physiological system with the functional capacity of that system, as the degree of complexity may differ between tasks and ecological conditions.

## Figures and Tables

**Figure A1:**
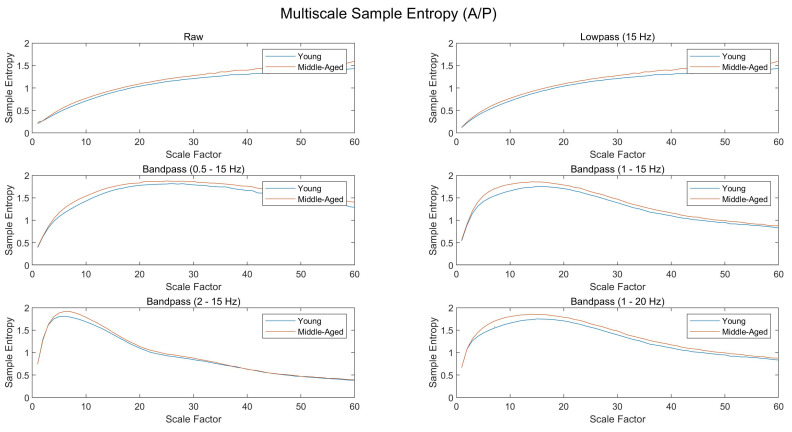
The effects of filter selection on CoP Multiscale Sample Entropy in the A/P direction.

**Figure A2:**
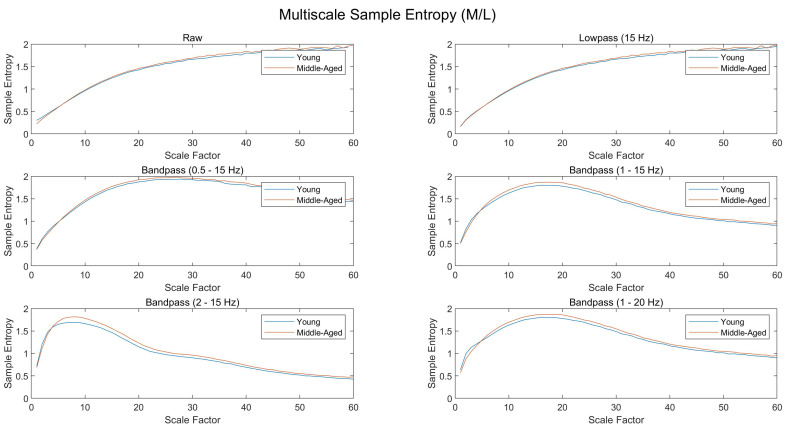
The effects of filter selection on CoP Multiscale Sample Entropy in the M/L direction.

**Figure A3:**
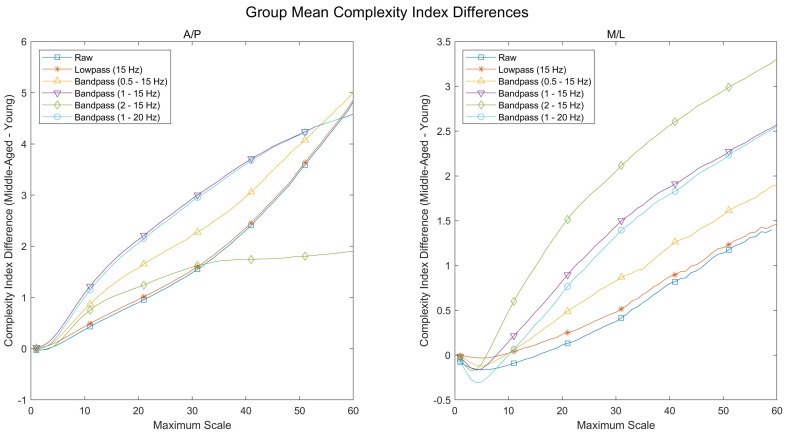
The effects of filter selection and maximum scale factor on group mean Complexity Index differences.

## Data Availability

All data used in the present study are available upon request to the corresponding author.

## Appendix A

**Table A1:** Repeated measures ANOVA—within-subjects effects (2.5–100 Hz; A/P).

Cases	Sphericity Correction	Sum of Squares	df	Mean Square	F	p	η^2^_p_
Condition	Greenhouse–Geisser	1041.267	1.537	677.425	12.189	<0.001	0.311
Condition × Age	Greenhouse–Geisser	348.059	1.537	226.440	4.074	0.034	0.131
Residuals	Greenhouse–Geisser	2306.501	41.502	55.576			

*Note.* Type III Sum of Squares.

**Table A2:** Repeated measures ANOVA—between-subjects effects (2.5–100 Hz; A/P).

Cases	Sum of Squares	df	Mean Square	F	p	η^2^_p_
Age	469.083	1	469.083	5.629	0.025	0.173
Residuals	2250.161	27	83.339			

*Note.* Type III Sum of Squares.

**Table A3:** Post hoc comparisons—condition (2.5–100 Hz; A/P).

		Mean Difference	SE	t	Cohen’s d	p_bonf_
Baseline	0 dB	6.995	1.717	4.073	0.933	<0.001
	−6 dB	7.648	1.717	4.453	1.020	<0.001
0 dB	−6 dB	0.652	1.717	0.380	0.087	1.000

*Note*. *p*-value adjusted for comparing a family of 3. *Note.* Results are averaged over the levels of: Age.

**Table A4:** Post hoc comparisons—age × condition (2.5–100 Hz; A/P).

		Mean Difference	SE	t	Cohen’s d	p_bonf_
Middle-Aged Baseline	Young Baseline	−0.661	2.787	−0.237	−0.088	1.000
	Middle-Aged 0 dB	3.867	2.470	1.565	0.516	1.000
	Young 0 dB	9.463	2.787	3.395	1.262	0.017
	Middle-Aged −6 dB	2.815	2.470	1.139	0.375	1.000
	Young −6 dB	11.820	2.787	4.241	1.576	<0.001
Young Baseline	Middle-Aged 0 dB	4.528	2.787	1.624	0.604	1.000
	Young 0 dB	10.124	2.386	4.242	1.350	0.001
	Middle-Aged −6 dB	3.476	2.787	1.247	0.463	1.000
	Young −6 dB	12.480	2.386	5.230	1.664	<0.001
Middle-Aged 0 dB	Young 0 dB	5.596	2.787	2.008	0.746	0.726
	Middle-Aged −6 dB	−1.052	2.470	−0.426	−0.140	1.000
	Young −6 dB	7.953	2.787	2.853	1.060	0.084
Young 0 dB	Middle-Aged −6 dB	−6.648	2.787	−2.385	−0.886	0.295
	Young −6 dB	2.357	2.386	0.987	0.314	1.000
Middle-Aged −6 dB	Young −6 dB	9.005	2.787	3.231	1.201	0.028

*Note. p*-value adjusted for comparing a family of 15.

**Table A5:** Repeated measures ANOVA—within-subjects effects (2.5–100 Hz; M/L).

Cases	Sum of Squares	df	Mean Square	F	p	η^2^_p_
Condition	668.720	2	334.360	10.328	<0.001	0.277
Condition × Age	210.751	2	105.376	3.255	0.046	0.108
Residuals	1748.120	54	32.373			

*Note.* Type III Sum of Squares.

**Table A6:** Repeated measures ANOVA—between-subjects effects (2.5–100 Hz; M/L).

Cases	Sum of Squares	df	Mean Square	F	p	η^2^_p_
Age	864.173	1	864.173	8.031	0.009	0.229
Residuals	2905.184	27	107.599			

*Note.* Type III sum of squares.

**Table A7:** Post hoc comparisons—condition (2.5–100 Hz; M/L).

		Mean Difference	SE	t	Cohen’s d	p_bonf_
Baseline	0 dB	3.905	1.495	2.612	0.515	0.035
	−6 dB	6.768	1.495	4.527	0.893	<0.001
0 dB	−6 dB	2.863	1.495	1.915	0.378	0.182

*Note. p*-value adjusted for comparing a family of 3. *Note.* Results are averaged over the levels of: Age.

**Table A8:** Post hoc comparisons—age × condition (2.5–100 Hz; M/L).

		Mean Difference	SE	t	Cohen’s d	p_bonf_
Middle-Aged Baseline	Young Baseline	4.513	2.817	1.602	0.595	1.000
	Middle-Aged 0 dB	4.302	2.151	2.000	0.568	0.757
	Young 0 dB	8.022	2.817	2.848	1.058	0.091
	Middle-Aged −6 dB	3.681	2.151	1.712	0.486	1.000
	Young −6 dB	14.369	2.817	5.101	1.896	<0.001
Young Baseline	Middle-Aged 0 dB	−0.211	2.817	−0.075	−0.028	1.000
	Young 0 dB	3.509	2.078	1.689	0.463	1.000
	Middle-Aged −6 dB	−0.832	2.817	−0.295	−0.110	1.000
	Young −6 dB	9.856	2.078	4.744	1.300	<0.001
Middle-Aged 0 dB	Young 0 dB	3.720	2.817	1.321	0.491	1.000
	Middle-Aged −6 dB	−0.621	2.151	−0.289	−0.082	1.000
	Young −6 dB	10.067	2.817	3.574	1.328	0.011
Young 0 dB	Middle-Aged −6 dB	−4.341	2.817	−1.541	−0.573	1.000
	Young −6 dB	6.347	2.078	3.055	0.837	0.052
Middle-Aged −6 dB	Young −6 dB	10.688	2.817	3.795	1.410	0.005

*Note. p*-value adjusted for comparing a family of 15.

**Table A9:** Repeated measures ANOVA—within-subjects effects (2.5–6 Hz; A/P).

Cases	Sphericity Correction	Sum of Squares	df	Mean Square	F	p	η^2^_p_
Condition	Greenhouse–Geisser	218.621	1.543	141.707	6.897	0.005	0.203
Condition × Age	Greenhouse–Geisser	91.130	1.543	59.069	2.875	0.080	0.096
Residuals	Greenhouse–Geisser	855.840	41.655	20.546			

*Note.* Type III Sum of Squares.

**Table A10:** Repeated measures ANOVA—between-subjects effects (2.5–6 Hz; A/P).

Cases	Sum of Squares	df	Mean Square	F	p	η^2^_p_
Age	265.391	1	265.391	7.601	0.010	0.220
Residuals	942.722	27	34.916			

*Note.* Type III Sum of Squares.

**Table A11:** Post hoc comparisons—condition (2.5–6 Hz; A/P).

		Mean Difference	SE	t	Cohen’s d	p_bonf_
Baseline	0 dB	3.113	1.046	2.976	0.661	0.013
	−6 dB	3.570	1.046	3.412	0.758	0.004
0 dB	−6 dB	0.456	1.046	0.436	0.097	1.000

*Note. p*-value adjusted for comparing a family of 3. *Note.* Results are averaged over the levels of: Age.

**Table A12:** Post hoc comparisons—age × condition (2.5–6 Hz; A/P).

		Mean Difference	SE	t	Cohen’s d	p_bonf_
Middle-Aged Baseline	Young Baseline	0.811	1.751	0.463	0.172	1.000
	Middle-Aged 0 dB	1.571	1.505	1.044	0.333	1.000
	Young 0 dB	5.466	1.751	3.122	1.160	0.039
	Middle-Aged −6 dB	1.085	1.505	0.721	0.230	1.000
	Young −6 dB	6.865	1.751	3.920	1.457	0.003
Young Baseline	Middle-Aged 0 dB	0.760	1.751	0.434	0.161	1.000
	Young 0 dB	4.655	1.454	3.202	0.988	0.034
	Middle-Aged −6 dB	0.274	1.751	0.157	0.058	1.000
	Young −6 dB	6.054	1.454	4.165	1.285	0.002
Middle-Aged 0 dB	Young 0 dB	3.895	1.751	2.224	0.827	0.441
	Middle-Aged −6 dB	−0.486	1.505	−0.323	−0.103	1.000
	Young −6 dB	5.294	1.751	3.023	1.123	0.053
Young 0 dB	Middle-Aged −6 dB	−4.381	1.751	−2.502	−0.930	0.221
	Young −6 dB	1.399	1.454	0.962	0.297	1.000
Middle-Aged −6 dB	Young −6 dB	5.780	1.751	3.301	1.227	0.023

*Note. p*-value adjusted for comparing a family of 15.

**Table A13:** Repeated measures ANOVA—within-subjects effects (2.5–6 Hz; M/L).

Cases	Sum of Squares	df	Mean Square	F	p	η^2^_p_
Condition	104.905	2	52.452	4.391	0.017	0.140
Condition × Age	67.668	2	33.834	2.832	0.068	0.095
Residuals	645.074	54	11.946			

*Note.* Type III Sum of Squares.

**Table A14:** Repeated measures ANOVA—between-subjects effects (2.5–6 Hz; M/L).

Cases	Sum of Squares	df	Mean Square	F	p	η^2^_p_
Age	503.394	1	503.394	11.418	0.002	0.297
Residuals	1190.337	27	44.087			

*Note.* Type III Sum of Squares.

**Table A15:** Post hoc comparisons—condition (2.5–6 Hz; M/L).

		Mean Difference	SE	t	Cohen’s d	p_bonf_
Baseline	0 dB	0.937	0.908	1.031	0.197	0.921
	−6 dB	2.653	0.908	2.922	0.557	0.015
0 dB	−6 dB	1.717	0.908	1.890	0.361	0.192

*Note. p*-value adjusted for comparing a family of 3. *Note.* Results are averaged over the levels of: Age.

**Table A16:** Post hoc comparisons—age × condition (2.5–6 Hz; M/L).

		Mean Difference	SE	t	Cohen’s d	p_bonf_
Middle-Aged Baseline	Young Baseline	4.396	1.769	2.485	0.924	0.239
	Middle-Aged 0 dB	1.689	1.306	1.293	0.355	1.000
	Young 0 Db	4.581	1.769	2.589	0.962	0.183
	Middle-Aged −6 dB	1.275	1.306	0.976	0.268	1.000
	Young −6 dB	8.428	1.769	4.765	1.771	<0.001
Young Baseline	Middle-Aged 0 dB	−2.707	1.769	−1.530	−0.569	1.000
	Young 0 dB	0.184	1.262	0.146	0.039	1.000
	Middle-Aged −6 dB	−3.121	1.769	−1.765	−0.656	1.000
	Young −6 dB	4.032	1.262	3.195	0.847	0.035
Middle-Aged 0 dB	Young 0 dB	2.891	1.769	1.635	0.607	1.000
	Middle-Aged −6 dB	−0.414	1.306	−0.317	−0.087	1.000
	Young −6 dB	6.739	1.769	3.810	1.416	0.005
Young 0 dB	Middle-Aged −6 dB	−3.306	1.769	−1.869	−0.694	1.000
	Young −6 dB	3.848	1.262	3.049	0.808	0.053
Middle-Aged −6 dB	Young −6 dB	7.154	1.769	4.044	1.503	0.002

*Note. p*-value adjusted for comparing a family of 15.

**Table A17:** Repeated measures ANOVA—within-subjects effects (8–12 Hz; A/P).

Cases	Sphericity Correction	Sum of Squares	df	Mean Square	F	p	η^2^_p_
Condition	Greenhouse–Geisser	29.760	1.520	19.584	18.565	<0.001	0.407
Condition × Age	Greenhouse–Geisser	7.794	1.520	5.129	4.862	0.020	0.153
Residuals	Greenhouse–Geisser	43.282	41.030	1.055			

*Note.* Type III Sum of Squares.

**Table A18:** Repeated measures ANOVA—between-subjects effects (8–12 Hz; A/P).

Cases	Sum of Squares	df	Mean Square	F	p	η^2^_p_
Age	8.691	1	8.691	4.938	0.035	0.155
Residuals	47.527	27	1.760			

*Note.* Type III Sum of Squares.

**Table A19:** Post hoc comparisons—condition (8–12 Hz; A/P).

		Mean Difference	SE	t	Cohen’s d	p_bonf_
Baseline	0 dB	1.214	0.235	5.160	1.147	<0.001
	−6 dB	1.267	0.235	5.387	1.197	<0.001
0 dB	−6 dB	0.053	0.235	0.226	0.050	1.000

*Note. p*-value adjusted for comparing a family of 3. *Note.* Results are averaged over the levels of: Age.

**Table A20:** Post hoc comparisons—age × condition (8–12 Hz; A/P).

		Mean Difference	SE	t	Cohen’s d	p_bonf_
Middle-Aged Baseline	Young Baseline	−0.166	0.393	−0.423	−0.157	1.000
	Middle-Aged 0 dB	0.737	0.338	2.178	0.696	0.507
	Young 0 dB	1.525	0.393	3.875	1.440	0.004
	Middle-Aged −6 dB	0.546	0.338	1.614	0.516	1.000
	Young −6 dB	1.822	0.393	4.631	1.721	<0.001
Young Baseline	Middle-Aged 0 dB	0.903	0.393	2.295	0.853	0.371
	Young 0 dB	1.691	0.327	5.173	1.597	<0.001
	Middle-Aged −6 dB	0.712	0.393	1.810	0.673	1.000
	Young −6 dB	1.988	0.327	6.082	1.878	<0.001
Middle-Aged 0 dB	Young 0 dB	0.788	0.393	2.002	0.744	0.737
	Middle-Aged −6 dB	−0.191	0.338	−0.564	−0.180	1.000
	Young −6 dB	1.085	0.393	2.758	1.025	0.111
Young 0 dB	Middle-Aged −6 dB	−0.979	0.393	−2.487	−0.924	0.229
	Young −6 dB	0.297	0.327	0.909	0.281	1.000
Middle-Aged −6 dB	Young −6 dB	1.276	0.393	3.243	1.205	0.027

*Note. p*-value adjusted for comparing a family of 15.

**Table A21:** Repeated measures ANOVA—within-subjects effects (8–12 Hz; M/L).

Cases	Sphericity Correction	Sum of Squares	df	Mean Square	F	p	η^2^_p_
Condition	Greenhouse–Geisser	23.204	1.558	14.891	17.533	<0.001	0.394
Condition × Age	Greenhouse–Geisser	4.503	1.558	2.890	3.403	0.054	0.112
Residuals	Greenhouse–Geisser	35.733	42.072	0.849			

*Note.* Type III Sum of Squares.

**Table A22:** Repeated measures ANOVA—between-subjects effects (8–12 Hz; M/L).

Cases	Sum of Squares	df	Mean Square	F	p	η^2^_p_
Age	12.948	1	12.948	5.255	0.030	0.163
Residuals	66.521	27	2.464			

*Note.* Type III Sum of Squares.

**Table A23:** Post hoc comparisons—condition (8–12 Hz; M/L).

		Mean Difference	SE	t	Cohen’s d	p_bonf_
Baseline	0 dB	0.847	0.214	3.961	0.754	<0.001
	−6 dB	1.238	0.214	5.792	1.102	<0.001
0 dB	−6 dB	0.391	0.214	1.831	0.348	0.218

*Note. p*-value adjusted for comparing a family of 3. *Note.* Results are averaged over the levels of: Age.

**Table A24:** Post hoc comparisons—age × condition (8–12 Hz; M/L).

		Mean Difference	SE	t	Cohen’s d	p_bonf_
Middle-Aged Baseline	Young Baseline	0.411	0.418	0.985	0.366	1.000
	Middle-Aged 0 dB	0.807	0.307	2.624	0.718	0.169
	Young 0 dB	1.298	0.418	3.108	1.155	0.044
	Middle-Aged −6 dB	0.737	0.307	2.396	0.656	0.301
	Young −6 dB	2.151	0.418	5.151	1.914	<0.001
Young Baseline	Middle-Aged 0 dB	0.396	0.418	0.948	0.352	1.000
	Young 0 dB	0.887	0.297	2.985	0.789	0.064
	Middle-Aged −6 dB	0.325	0.418	0.780	0.290	1.000
	Young −6 dB	1.740	0.297	5.857	1.548	<0.001
Middle-Aged 0 dB	Young 0 dB	0.491	0.418	1.175	0.437	1.000
	Middle-Aged −6 dB	−0.070	0.307	−0.229	−0.063	1.000
	Young −6 dB	1.344	0.418	3.219	1.196	0.032
Young 0 dB	Middle-Aged −6 dB	−0.561	0.418	−1.344	−0.499	1.000
	Young −6 dB	0.853	0.297	2.872	0.759	0.087
Middle-Aged −6 dB	Young −6 dB	1.414	0.418	3.387	1.259	0.020

*Note. p*-value adjusted for comparing a family of 15.

## Appendix B

A sensitivity analysis was performed to examine the effects of filter selection on Multiscale Sample Entropy (MSE). First, MSE was computed for the CoP data using six filtering approaches: raw (i.e., no filter), a lowpass filter at 15 Hz, a bandpass filter at 0.5–15 Hz, a bandpass filter at 1–15 Hz, a bandpass filter at 2–15 Hz, and a bandpass filter at 1–20 Hz. These results are shown below for the CoP data in the A/P and M/L directions in Figure A1 and Figure A2, respectively.

Once the MSE was computed, Complexity Indices were computed for each of the six filtering techniques across a range of scale factors. Group mean differences were then computed and plotted as a function of maximum scale (Figure A3). Note that a maximum scale of 40 was used in the present study based on recommendations by Yentes et al. [12] to compute Sample Entropy on no fewer than 200 samples when using the recommended *m* criterion of 2. As each CoP signal analyzed in the present study contained approximately 8000 samples, the coarse-graining procedure would yield approximately 200 samples per signal at scale 40.

In comparing filtering approaches, we selected the 1–15 Hz bandpass filter in order to remove low-frequency nonstationarities as well as to retain only those frequencies which are physiologically feasible [17]. This filtering approach also appeared to maximize age-related differences while yielding consistent trends across CoP directions (Figure A3).
